# A method for detecting ureteral stent encrustations in medical CT images based on Mask-RCNN and 3D morphological analysis

**DOI:** 10.3389/fphys.2024.1432121

**Published:** 2024-08-29

**Authors:** Hongji Hu, Minbo Yan, Zicheng Liu, Junliang Qiu, Yingbo Dai, Yuxin Tang

**Affiliations:** Department of Urology, Fifth Affiliated Hospital of Sun Yat-sen University, Zhuhai, Guangdong, China

**Keywords:** artificial intelligence, ureteral stent encrustation, medical imaging, neural network, stone detection

## Abstract

**Objective:**

To develop and validate a method for detecting ureteral stent encrustations in medical CT images based on Mask-RCNN and 3D morphological analysis.

**Method:**

All 222 cases of ureteral stent data were obtained from the Fifth Affiliated Hospital of Sun Yat-sen University. Firstly, a neural network was used to detect the region of the ureteral stent, and the results of the coarse detection were completed and connected domain filtered based on the continuity of the ureteral stent in 3D space to obtain a 3D segmentation result. Secondly, the segmentation results were analyzed and detected based on the 3D morphology, and the centerline was obtained through thinning the 3D image, fitting and deriving the ureteral stent, and obtaining radial sections. Finally, the abnormal areas of the radial section were detected through polar coordinate transformation to detect the encrustation area of the ureteral stent.

**Results:**

For the detection of ureteral stent encrustations in the ureter, the algorithm’s confusion matrix achieved an accuracy of 79.6% in the validation of residual stones/ureteral stent encrustations at 186 locations. Ultimately, the algorithm was validated in 222 cases, achieving a ureteral stent segmentation accuracy of 94.4% and a positive and negative judgment accuracy of 87.3%. The average detection time per case was 12 s.

**Conclusion:**

The proposed medical CT image ureteral stent wall stone detection method based on Mask-RCNN and 3D morphological analysis can effectively assist clinical doctors in diagnosing ureteral stent encrustations.

## 1 Introduction

Since the introduction of the ureteral stent in 1978 by Finney (1978). The ureteral stent is a common implanted device placed by doctors inside the patient’s body after surgery for urinary tract stone (Chew and Lange, 2009). It serves to protect and restore kidney function, drain renal pelvis fluid, and relieve temporary blockages caused by ureteral inflammation and edema, prevent postoperative urine leakage and ureteral strictures (Tomer et al., 2021). However, as the duration of stent placement in the body increases, it may lead to the formation of stones on the inner and outer walls of the stent (Dyer et al., 2002; Saadi et al., 2023). According to the report in J Urol in 2021, the incidence of encrustations in ureteral stents can be as high as 13%, and the incidence gradually increases with the prolonged duration of stent placement (Chew and Lange, 2009). Accumulation of encrustations may make it difficult to remove the stent for patients, subsequently. For patients with relatively smooth ureteral stents and less severe encrustations, stent removal can be performed in the outpatient department. However, in cases the encrustations in the ureteral stent are severe, extracorporeal shock wave lithotripsy may be necessary before removing the intrarenal stent, and in some cases, surgical stone fragmentation may be required before stent removal. If patients do not undergo comprehensive imaging examinations and doctors are unaware of the situation of the ureteral stent in the body, attempting to remove the stent in the outpatient department without prior assessment can lead to stent removal failure due to the influence of ureteral stent encrustations, increasing the patient’s pain and burden.

However, identifying ureteral stent encrustations is still a challenge. Since its invention in the 1970s, CT have established it as an indispensable tool in the practice of medicine (McCollough and Rajiah, 2023). As a routine examination method, has the advantages of non-invasiveness, fast imaging, and high image resolution, making it an important means of screening. A clinical study show that traditional CT imaging methods lack sensitivity, specificity, positive predictive value, negative predictive value, and accuracy (Tang and Attwell-Heap, 2011). Moreover, Saadi A et al. used the KUB encrusted stent scoring system and FECal grading system to predict the complexity of ureteral stent removal surgery (Saadi et al., 2023). None of the above can fail to identify ureteral stent encrustations quickly and accurately.

With the rapid improvement of computer hardware performance, deep learning methods have emerged and demonstrated powerful capabilities in image processing tasks. Shen et al. summarized various medical image analysis methods (Chan et al., 2020). The Microsoft team, led by He, proposed a residual network architecture, effectively addressing the problem of neural network gradient dispersion. Prathiba et al. integrated various network structures to construct a deep residual fully convolutional network (FCN), which significantly improves the automatic segmentation of melanoma in dermatoscopy images (He et al., 2016). The aforementioned technological advancements have effectively improved the radiological diagnosis of diseases such as pulmonary nodules, but there is a lack of research in the detection of ureteral stent encrustations.

Early identification and prevention encrustation are one of the most effective measures to treat the complications of encrustation. A new method for identifying ureteral stent encrustations is developed, allowing doctors to more accurately identify the presence of ureteral stent encrustations and assess their severity before removing the ureteral stent from the patient, optimizing the stent removal process. Ultimately the model could benefit patients.

## 2 Materials and methods

All experiments in this paper were conducted in the same experimental environment, with a computer configuration of Intel(R) Core i5-9400 CPU at 2.90 GHz, 16 GB of memory, and an NVIDIA GeForce RTX 3080 graphics card. The software platform is based on the Python three language and the Porch framework.

**FIGURE 1:**
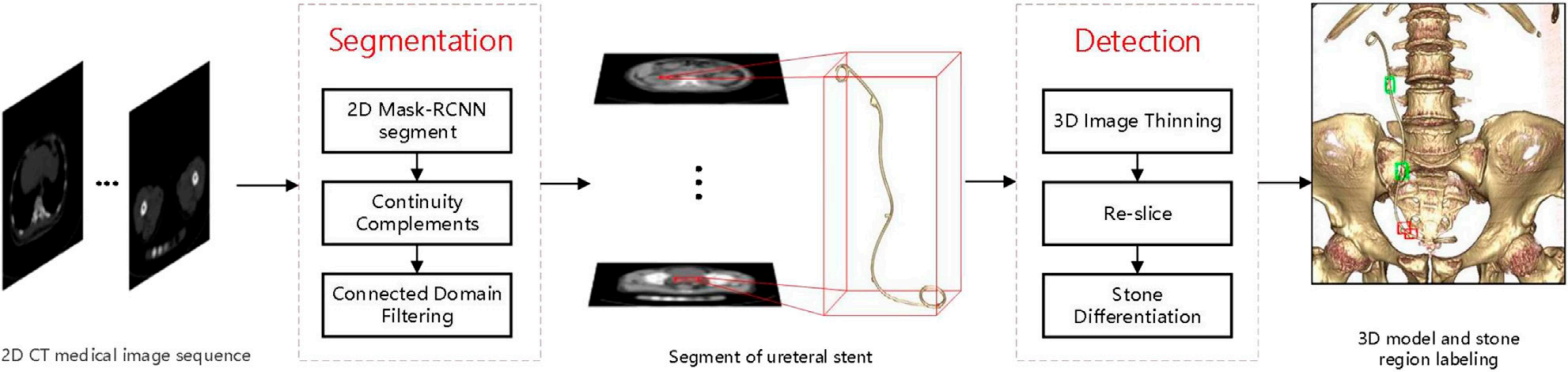
Flow chart of the method.

A medical CT image-based method is proposed for detecting ureteral stent encrustation in ureteral stents using Mask-RCNN (He et al., 2017) and 3D morphological analysis. The method can segment the ureteral stent and differentiate residual stones and ureteral stent encrustation near the stent. Firstly, a neural network is used to detect the region of the ureteral stent in the input 2D CT medical image sequence, and the results of the coarse detection are completed and connected by filling in the gaps and filtering the connected domains based on the continuity of the ureteral stent in 3D space to obtain the 3D segmentation result. Secondly, the segmentation result is analyzed and detected based on 3D morphology analysis. The center line is obtained by 3D image thinning, and the ureteral stent is re-sliced and radial sections are obtained by fitting and derivation. Finally, the abnormal area of the re-sliced section is detected by polar coordinate transformation to detect the encrustation area of the ureteral stent. The overall algorithm flowchart is shown in Figure 1.

### 2.1 Ureteral stent segmentation

#### 2.1.1 2D neural network detection

Mask-RCNN is an important breakthrough in the development of two-stage object detection networks. In addition to introducing simple masks to achieve instance segmentation, the most critical aspect is the introduction of the feature pyramid network (FPN). The FPN network adds up features layer by layer from bottom to top, generating new four-layer feature maps that fuse multiple depth information. This structure can improve accuracy to some extent when added to many networks, especially for small target objects. The output channel number of each layer of FPN is usually set to 256, because it fuses depth features layer by layer from the bottom with enough information, and will not cause a decrease in accuracy due to excessive dimensionality reduction and information loss, while also reducing the complexity of the network.

The atrous spatial pyramid pooling (ASPP) layer proposed in the DeepLab series by Google (Chen et al., 2017a; Chen et al., 2018; Chen et al., 2017b) is an effective network structure for small target semantic segmentation tasks. It increases the receptive field of each shared convolutional kernel, avoids the pooling downsampling process, and fills the lost contextual information during feature compression. ASPP structure is to use dilated convolutions with different dilation rates to obtain compressed features at different receptive field ranges for the same input feature, and then concatenate the results containing different scale information. Finally, a convolutional layer is used to reduce the channel dimension of the concatenated result.

#### 2.1.2 Multi-Task learning

Multi-Task learning is a machine learning method that integrates multiple related tasks using shared representation technology, fully utilizing the correlation information between tasks to improve the generalization performance of single-task learning network. Joint learning of different tasks can effectively mine different feature correlation information in images, improving the performance and generalization of the model.

**FIGURE 2:**
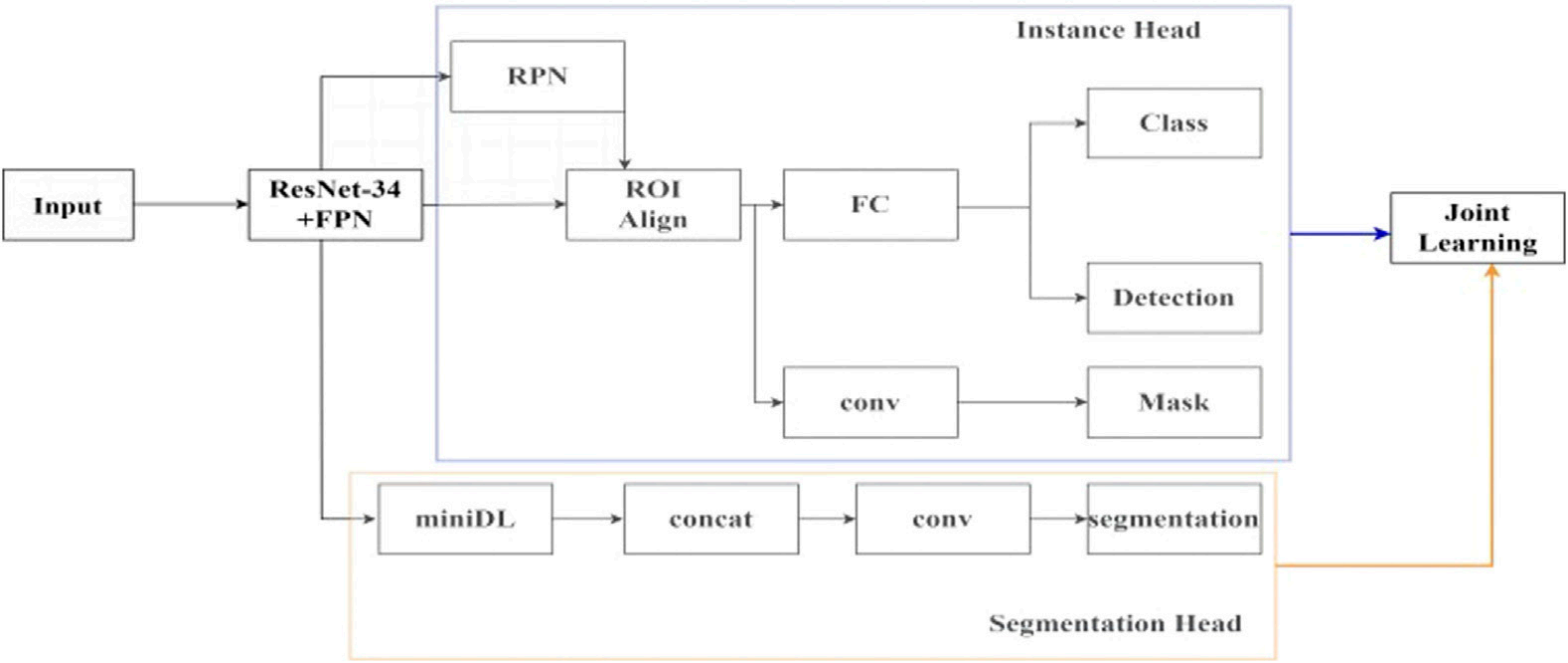
The framework of joint learning neural network.

In this study, the idea of joint learning is integrated into the Mask-RCNN network, and a semantic segmentation branch is added. The overall joint learning neural network framework is shown in Figure 2.

In the feature extraction network module, the ResNet34 backbone network is used, and the features of the last four layers of different depths are taken. Then, the FPN layer is added to add up the features layer by layer from bottom to top to generate new four-layer feature maps that fuse multiple depth information, which can achieve good detection results for small targets. In the ResNet34-FPN encoder, based on the extraction of features at different depths by ResNet34, the FPN layer is used to exchange information between features at different depths and unify them into 256 channels, removing redundant information while avoiding complete loss of deep semantic information, and without significantly increasing the complexity of the network. Two branches are connected after the feature extraction network. The first branch is for instance segmentation task, which first uses the region proposal network (RPN) to select the regions of interest, and then performs bounding box regression, classification, and mask segmentation within the regions of interest to obtain the detection result of the ureteral region. The second branch is for semantic segmentation task, using a simplified version of the ASPP structure. In the decoder of the semantic segmentation task, the features of different scales with the number of four layers of output channels of 256 are obtained through the miniDL module, so it could be capturing contextual information from a larger receptive field, which reduces the false positive rate. Unlike the binary mask segmentation only within the detection box in the instance segmentation task, this task starts from the overall image, analyzes and learns high-level semantic features, and realizes pixel-level classification of the ureteral stent. Finally, the loss functions of the two sub-tasks are weighted and output, and joint learning affects the parameters of the two sub-tasks together.

#### 2.1.3 3D connected domain filtering

Due to the overlapping density ranges of urinary tract stones and ureteral stents in the human body, i.e., similar CT values in CT images, neural network detection on 2D images alone will result in partial small stones being falsely detected. Hence, the connectivity of the ureteral stent in 3D space is an important condition for excluding the remaining scattered stones.

Connected component labeling is the basis for all binary image analysis. It marks the target pixels in the binary image, allowing each separate connected region to form a labeled block. Further, we can obtain geometric parameters such as contours, bounding rectangles, centroids, and moments for these blocks. In 3D discrete space, there are three types of adjacency relationships, 6-adjacency, 18-adjacency, and 26-adjacency.

A connected component is a pixel set composed of adjacent pixels with the same pixel value. By using these two conditions, connected regions can be found in the image, and for each connected region found, a unique label is assigned to distinguish it from other connected regions. The two-pass scanning method is used, and the algorithm is as follows in Table 1.

**TABLE 1:** Two-pass scanning method.

Input: 3D data matrix
Output: 3D connected domain sequences
Step 1: The first scan Access the current voxel B (x, y, z), if B (x, y, z) = 1: a, If the label values in the neighborhood of B (x, y, z) are all 0, then assign a new label to B (x, y) label + = 1, B (x,y,z) = label b, If there are voxels Neighbors with voxel values >1 in the neighborhood of B (x, y, z): 1) Assign the minimum value in Neighbors to B (x, y, z) B (x,y,z) = min{Neighbors} 2) Record the equality relationship between the values (labels) in Neighbors, indicating that these values (labels) belong to the same connected region
Step 2: The second scan: Access the current voxel B (x, y, z), if B (x, y, z) > 1: Find the minimum label value in the equality relationship with label = B (x, y, z), and assign it to B (x, y, z)
Step 3: Complete the scan, voxels with the same label values in the image consisted of the same connected region

**FIGURE 3:**
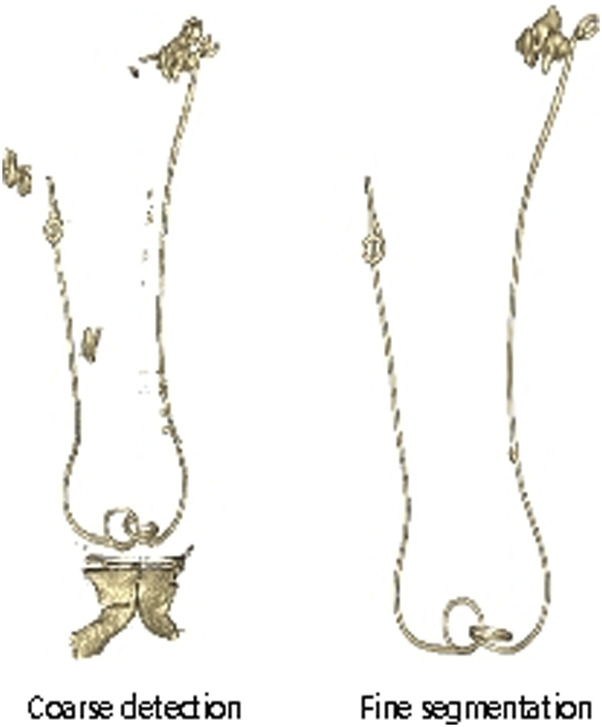
Comparison of segmentation results before and after filtering in connected domain.

The detection results of the 2D sequence are combined to obtain the rough detection results of the 3D ureteral stent. After connected component filtering, the final 3D segmentation results of the ureteral stent are obtained, as shown in Figure 3.

### 2.2 Ureteral stent encrustation detection

#### 2.2.1 3D image refinement

The simplified 1D curve describes the original 3D object in a linear representation, which is generally referred to as a centerline or curve-skeleton. The curve-skeleton is defined as follows:

Set up a collection $X\in Z^{n}$ , B is a closed unit ball, then the skeleton SK(X) of set X is composed of a series of skeleton subsets SK(X)_r, $SK\left(X\right)=\underset{r\geq 0}{\cup }SK_{r}\left(X\right)$ ,which $SK_{r}=\underset{k\geq 0}{\cap }\left[{\left(X⊗rB\right)}_{kB}\right]$ .

This paper adopts the centerline of the 3D ureteral stent obtained by the template-based algorithm in the topological refinement method (Lee et al., 1994). Starting from the boundary of the shape and moving inwards, the position of the central skeleton is gradually searched. The basic idea is to uniformly peel off the boundary points of the shape layer by layer, and the remaining innermost part that cannot be further peeled off forms the skeleton of the shape. The template-based algorithm applies a refinement operator to each point on the image or object in the order of scan lines, and it makes a judgment by examining the neighborhood of point p. This refinement operator can be viewed as a Boolean function, with its input being the value of p and its neighboring elements, and its output being the new value of p. The entire process is carried out repeatedly, with all points being scanned each time, identifying and removing points that can be deleted, until no more points can be removed. This algorithm is based on the neighborhood of a given point, typically using an 8-neighborhood (3 × 3 template) in 2D images and a 26-neighborhood (3 × 3 × 3 template) in 3D space.

**FIGURE 4:**
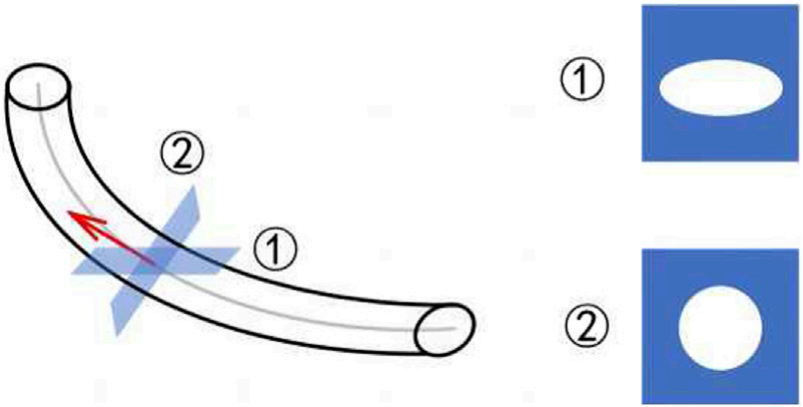
Visualization of the centerline of 3D ureteral stent.

The 3D visualization of the skeleton graph is shown in Figure 4A, where the blue area represents the original 3D segmentation result of the ureteral stent, and the red line represents the obtained centerline. To observe the accuracy of the skeleton extraction more clearly, projection images in the *x* and *y* directions are plotted, as shown in Figure 4B.

#### 2.2.2 3D repeat sections

**FIGURE 5:**
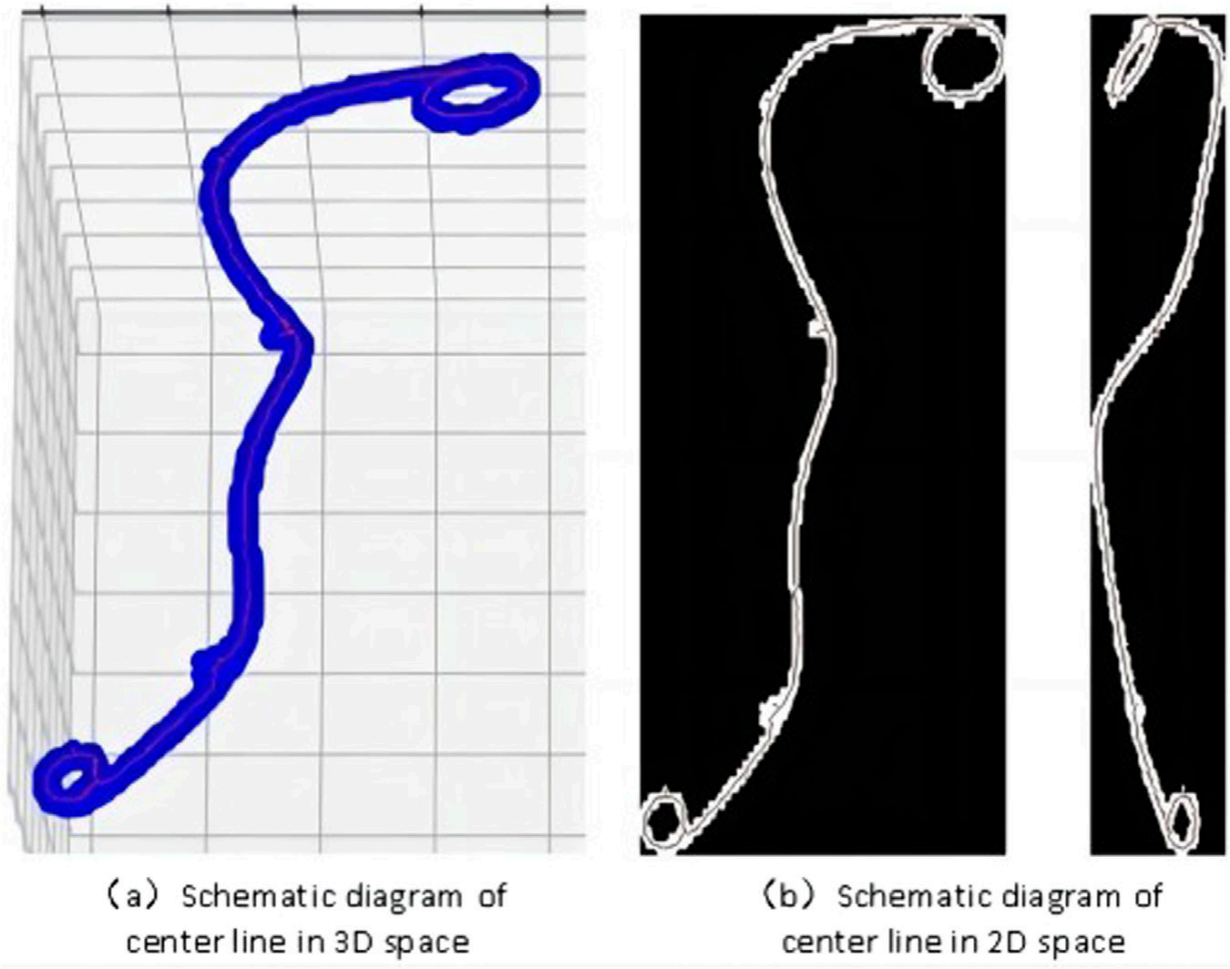
3D reslice schematic.

Figure 5 shows the complex and variable morphology of the ureteral stent in the human body, which can easily tilt. However, CT imaging only slices at fixed intervals in one direction, as shown in ①, causing the cross-section to become an irregular circle, which can interfere with the assessment of ureteral stent encrustation. In this case, it is necessary to re-slice the entire ureteral stent, resulting in a circular shape in the direction of the pipe diameter, as shown in ②. This allows for effective assessment of any abnormalities in the ureteral stent. The re-slicing steps of the ureteral stent are as follows.

Step 1: Use the B-spline method to fit the centerline of the ureteral stent, as shown by the gray line in Figure 5.
Step 2: Take the derivative of the fitted curve to obtain the tangent at each center point, as shown by the red line in Figure 5.
Step 3: Use the tangent as the normal vector to calculate the cross-section corresponding to each center point in the direction of the pipe diameter.

After completing the re-slicing, the cross-sectional area of each re-slice is calculated. The area sequence is then normalized and sorted. If the area values before and after in the sequence exceed a threshold, they are used as boundary points. The points with area values greater than the boundary points are divided into the stone area, which can preliminarily identify abnormal sections of the ureter.

#### 2.2.3 Differentiating residual stones from ureteral stent encrustations

**FIGURE 6:**
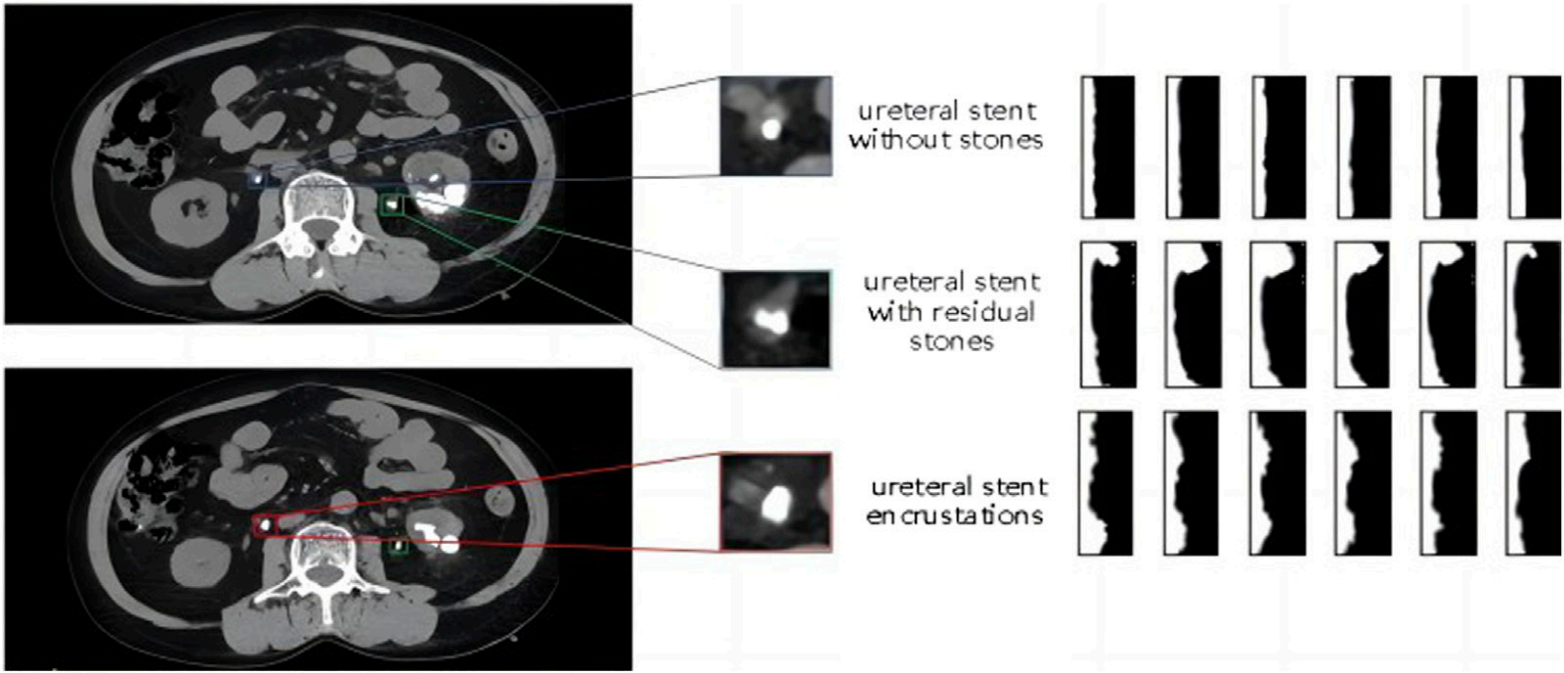
Comparison diagram of the morphology and polar coordinate transformation characteristics of three types of ureteral stents.

Polar coordinate transformation refers to converting Cartesian coordinates into polar coordinates, with the two axes being the angle θ and the major axis. The position of a point is represented by the angle with the horizontal direction from the origin and the distance to the origin. It is challenging to describe circular objects when distinguishing features. Therefore, the polar coordinate transformation method is often used to map circular objects to rectangular features for analysis in the defect detection of circular objects. In this study, there are three categories to differentiate, including ureteral stents without encrustations, residual stones that have fallen and come into contact with the stent and ureteral stent encrustations. All of them are small targets with similar shapes and are difficult to distinguish directly. As shown in the shape comparison in Figure 6, the left side shows the enlarged contrast of the original single CT image, and the right side shows the feature map after the polar coordinate transformation of a continuous segment corresponding to the region.

**FIGURE 7:**
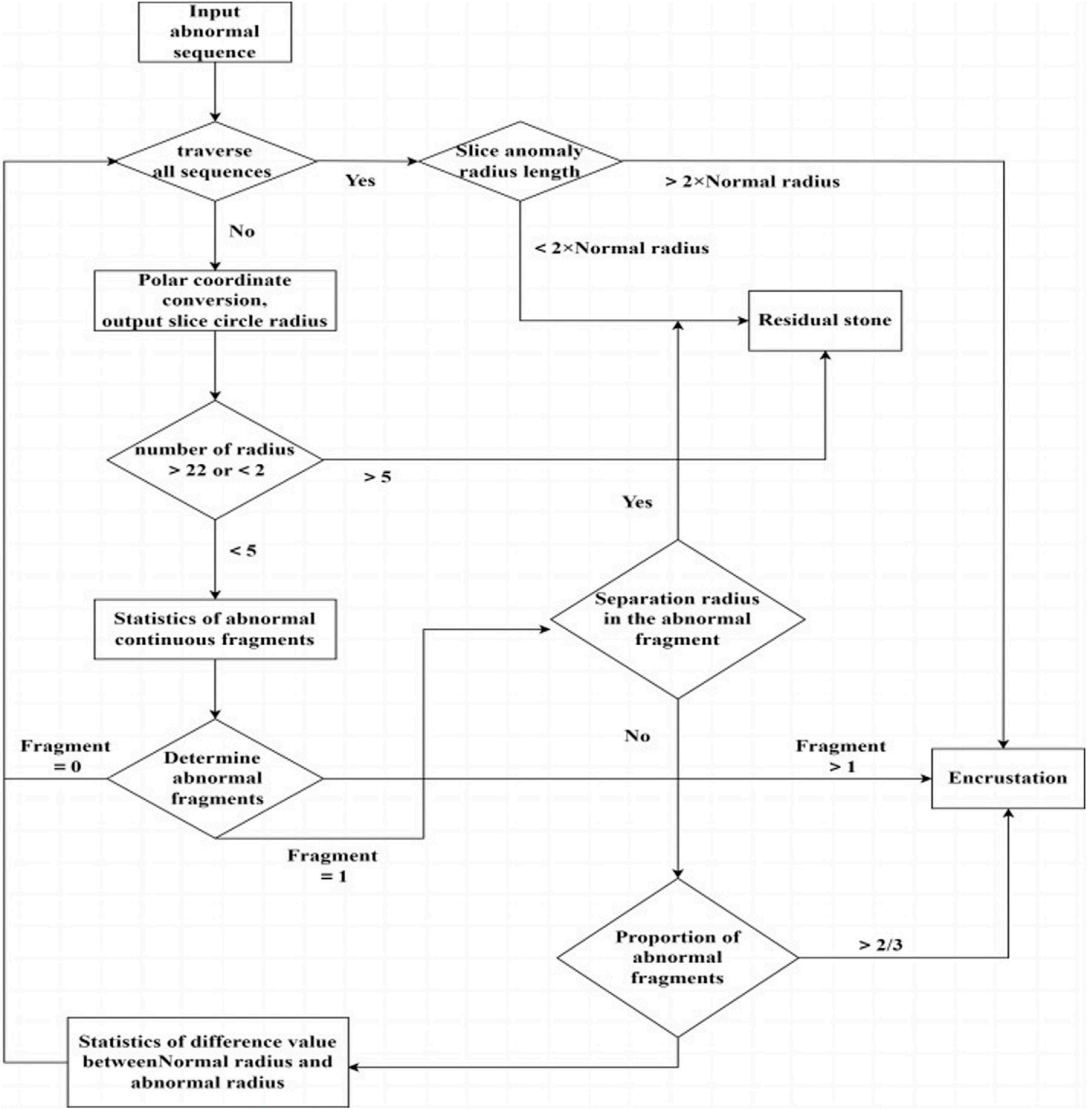
Flow chart of algorithm to distinguish residual stones and encrustations.

The image after polar coordinate transformation can be interpreted as a continuous radius sequence for a circular shape. From the top image in Figure 6, it can be observed that the ureteral stent without stones tends to be a standard circle, with a stable and not very noticeable fluctuation in the radius. The stent with residual stones shows a concentration of abnormally increased radius in a continuous segment, indicating that the residual stones are in contact with the stent, but not closely, and with a larger increase in radius, which does not conform to the growth pattern of ureteral stent encrustations. On the other hand, ureteral stent encrustations show intermittent increases in the entire radius sequence, consistent with the growth pattern of encrustations around the stent. The detailed algorithm flow for differentiating residual stones and ureteral stent encrustations is shown in Figure 7.

## 3 Results

### 3.1 Data summary indicates

This study was approved by the Ethics Review Committee of the Fifth Affiliated Hospital of Sun Yat-sen University. The CT dataset for detecting ureteral stent encrustations were collected by clinical doctors in the urology department of the Fifth Affiliated Hospital of Sun Yat-sen University, with a total of 222 patient cases, including 67 positive cases and 155 negative cases. The data have been obtained with the informed consent of the patients.

For the ureteral stent segmentation task, clinical doctors performed semantic segmentation labeling on the ureteral stent area of 21^−ΔΔCT^ datasets, totaling 2985 images. These were divided into a training set and a validation set in a 4:1 ratio. In addition, 132 cases without semantic labeling were selected as the test set, totaling 16,262 images.

For encrustation detection, clinical doctors further conducted detailed stone area annotations, distinguishing between residual stones and encrustations. Residual stones generally refer to stones that have fallen from the kidney area to the ureteral area after lithotripsy and are in contact with the ureteral stent, without affecting stent removal. On the other hand, encrustations refer to stones that grow on the outer wall of the ureteral stent as time passes while the stent is in place in the body. Among the 222 patient cases, there were 87 instances of attached stones and 99 instances of residual stones.

### 3.2 Ureteral stent segmentation in different neural network models

For the small object detection problem, comparative tests were conducted on different basic object detection frameworks, including semantic segmentation models Deeplab v3+ and BiseNet, and object detection models Mask-RCNN, YOLO v5, and CenterNet. From Table 4, it can be seen that Mask-RCNN has a loss rate of 1.8% and a false alarm rate of 2.9% in testing, performing the best among all models. The comparison results of the missed detection and false alarm rates on the 16,262 test set images are shown in Table 2.

**TABLE 2:** The segmentation and detection results of different neural network models.

Type of task	Network model	Loss (%)	Mischeck rate (%)
Semantic Segmentation	BiseNet	4.3	2.7
	DeepLab v3+	4.1	3.1
Object Detection	Mask-RCNN	1.8	2.9
	yolo v5	2.8	6.3
	CenterNet	2.7	4.9

### 3.3 Effect of joint learning and connected domain filtering on the segmentation results

The Intersection over Union (IoU) measure is a standard for assessing the accuracy of visual methods, commonly used to evaluate the performance of techniques such as object detection and semantic segmentation. The commonly used metric for object detection tasks is Mean Average Precision (MAP). mAP means the average AP for all classes after computing the AP for each individual class. The comparison of joint learning and the validation set metrics for each subtask is shown in Table 3. From the training indicators, the mIOU for the semantic segmentation subtask increased from 0.836 to 0.861, and the mAP for the object detection subtask increased from 0.842 to 0.855. Combination learning can effectively improve the accuracy of both subtasks.

**TABLE 3:** Comparison of the joint-learning segmentation results.

Type of task	mAP	miaou
Semantic Segmentation	—	0.836
Object Detection	0.842	—
Combination Learning	0.855	0.861

Further analysis of the missed detection and false alarm situations in actual cases was conducted to verify the effectiveness of the connected component filtering method. The test results in 132 cases are compared in Table 4. It can be observed that the introduction of 3D connected components can significantly reduce the false alarm rate for ureteral stents, with the number of cases with false alarms decreasing from 69 to just 1.

**TABLE 4:** Comparison of the segmentation results for connected domain filtering.

Error	Cause of error	Combination learning (case)	Combination learning + 3D connected domain filtering (case)
False Detection	Close stent small stones	29	0
	Complex large stone at the distal end of the DJ tube	19	0
	Bone false alarm	20	1
	False detection of other tubular objects	1	0
	Total	69	1
Leak Detection	Missed detection of the complex part at the distal end of the DJ tube	5	5
	Missed detection at the sharp bend of the DJ tube	3	2
	Total	8	7

### 3.4 Analysis of the detection results of ureteral stent encrustations

For the task of detecting ureteral stent encrustations, as there were no previous studies in this direction for comparison, the proposed experimental method was tested in 222 cases. The confusion matrix for the algorithm validation in 186 cases of residual stones and ureteral stent encrustations is shown in Table 5, with an accuracy of 79.6%.The confusion matrix of sex judgments in 222 cases is shown in Table 6, with an accuracy of 87.3%.

**TABLE 5:** Confusion matrix of residual stone detection results.

	Real stone	Real residual stone
Predicting stones	78	29
Predicting residual stone	9	70

**TABLE 6:** Yin judgment confusion matrix.

	Positive	Negative
Predicted positive	54	15
Predicted negative	13	140

The final algorithm validation was conducted in 222 cases for the two tasks proposed in this paper. The accuracy of ureteral stent segmentation reached 94.4%, and the accuracy of differentiating between positive and negative cases reached 87.3%. The average detection time per case was 12 s. It can be observed that the proposed method can effectively assist hospital doctors in diagnosing ureteral stent encrustations.

## 4 Discussion

Ureteral stents are commonly used postoperatively in urological stone surgery, and encrustations may form on the surface and/or within the lumen of the stent after insertion (Chew and Lange, 2009; Zhao et al., 2016). People always focus on the material and structure of ureteral stents to prevent complications, such as using “suture stents” to reduce symptoms associated with ureteral stents; Vogt et al. (2015) introduced an improved polyurethane double-J stent to alleviate the discomfort caused by ureteral stents, all of the above often overlook the complications themselves. No matter how to delay the formation of encrustations, with the increase of indwelling time, the encrustation is inevitable (Lange et al., 2015), so the key is to early and more accurately detect encrustation. Meanwhile endourological management of ureteral stent encrustation remains technically and strategically challenging (Tsaturyan et al., 2023). Multi-angle and multiaspect strategies are generally required. A systematic review on behalf of the EAU YAU Urolithiasis Group revealed that 27% of the encrusted stents require a combined surgery, followed by 24% of URS alone or 19% of SWL alone as a single surgery (Massella et al., 2023; Juliebø-Jones et al., 2021). The combined model enables identification of suspicious encrustation with high accuracy, which assists urologists to distinguish encrustations from the residual stones,and in taking a single timely surgery and prevents further aggravation (Lombardo et al., 2022; Geraghty et al., 2023).

Artificial intelligence (AI) is being increasingly integrated into scientific discovery to augment and accelerate research, including geometric deep learning (Wang et al., 2023). Abdolmanafi et al., for example, utilized a deep-learning CNN in the classification of coronary artery optical coherence tomography (OCT) images in patients with Kawasaki disease (Ng et al., 2016). Zhang et al. (2023) uesd an artificial intelligence network-guided signature for predicting outcome and immunotherapy response in lung adenocarcinoma patients based on 26 machine learning algorithms. For ureteral stent encrustation, Liu et al. (2024) predicted the risk of encrustation in patients with calculi based on their biochemical data; Qiu et al. (2023) used medical imaging-based techniques to preliminarily identify ureteral stent encrustation. These are just simple explorations into whether encrustations exist, and cannot accurately and efficiently identify them.

Therefore, A computer vision method for detecting encrusted stones on ureteral stents to assist doctors in judgment is crucial. Firstly, there are strict criteria for distinguishing between the ureteral stent area and non-ureteral stent area, which can be accurately segmented and reconstructed to create a 3D model of the ureteral stent, making it more convenient for doctors to view compared to 2D images. As for the severity of the stones, distinguishing residual stones and encrustations, which are subjective and less strict in nature, computer vision can accurately locate the abnormal areas, provide preliminary diagnostic results, and then have the doctors confirm the case results through film reading. There have been no reports in the literature so far on a medical CT imaging method for detecting encrusted stones on ureteral stents that combines Mask-RCNN with 3D morphological analysis.

We use a deep learning model, Mask-RCNN, to accurately identify ureteral stents in CT images and analyze if there are encrustations around them. As shown in Table 4, Compared to other conventional neural network models, our model has lower loss and false positive rates in both semantic segmentation and object detection models. Additionally, as shown in Table 5, by employing a joint learning approach, we were able to increase the Intersection over Union (IoU) to 85.5% and improve the Mean Average Precision (MAP) to 86.1%.Compared with the common methods for identifying encrustations on ureteral stents, such as CT and DR imaging examinations, the proposed method has higher accuracy and also improves the efficiency of clinical doctors. Then, compared to the currently recognized gold standard of ureteroscopy, our method is simple, fast, and non-invasive.

Compared to methods based on mathematical morphology, edge detection, and thresholding, the segmentation method based on deep learning neural networks has the advantage of automatically extracting image information features, iteratively optimizing, and utilizing the network’s non-linear characteristics for boundary segmentation during training. However, the limitation of this method is that when facing new image features, the network model needs to be retrained, and the parameter tuning process is relatively complex. Moreover, judging image features locally through convolution may not ensure good connectivity in 3D segmentation. In the end, we use 2D object detection to segment the ureteral stent area in CT images, introduce the idea of vessel tracking to complete the center points, effectively connect difficult-to-segment parts, and then filter the 3D connected regions to remove similar ureteral stents and stones.

For the detection method of ureteral stent encrustations, it is more of a mathematical morphological analysis, summarizing and classifying the 2D morphology of ureteral stents obtained from re-slicing. The 3D reconstruction of the ureteral stent actually involves a process that starts with the input of 2D CT image sequences, followed by 2D deep learning-based segmentation of the ureteral stent, reconstruction of the stent’s cross-sectional sequence to obtain rough 3D data, and then uses 3D connected domain screening to remove stones that are not attached to the ureteral stent, ultimately achieving a precise 3D segmentation result of the ureteral stent. The ureters have a complex shape and are surrounded by stones with similar CT values, making them difficult to directly use threshold-based segmentation to distinguish the stent from the stones. Moreover, since the ureteral stent is a connected object, introducing connected domain screening can effectively remove isolated stones. This reconstruction method can also be applied to other connected organs, such as vessels, which have similar characteristics. The main purpose of the 3D repeat sections in our article is to re-slice the reconstructed data after completion, making the sections used for stone detection perpendicular to the centerline of the stent. This has significant benefits in judging abnormalities in inclined and complex tubular structures. Because encrustations and residual stones will both increase the diameter of the ureteral stent, it is impossible to directly use the original *Z*-axis slices to detect the presence of encrustations by analyzing the inner circle radius abnormality. Instead, re-slicing is necessary to obtain sections perpendicular to the centerline for further analysis, and the detailed judgment process can be seen in the supplementary explanation of the previous comment. It is possible to accurately measure the increase in radius of encrustations growing on ureteral stents using a computer, reducing the workload of doctors and improving work efficiency. At the same time, abnormal radius areas can be selected and analyzed before being provided to doctors for auxiliary judgment.

There are also some limitations in this study. Due to the limited sample size, we were unable to validate our model across multiple centers. In the future, we plan to use our equipment to validate it in different scenarios across multiple hospitals. The method based on deep learning needs to be further validated on a more extensive dataset. Therefore, transfer learning will be used to improve the model’s generalization to patients with urological stones in future work. Additionally, judgement metrics from deep learning outputs will be used to develop a new evaluation standard to quantify the severity of encrustations. In addition, interpretability is very important in medical image analysis applications. Enhancing the interpretability of deep neural networks in various tasks of medical image analysis has always been a challenge, and further research in this area is needed.

## 5 Conclusion

A method for detecting encrustations on ureteral stents in medical CT images is proposed, which integrates Mask-RCNN with 3D morphological analysis. This method can effectively detect and differentiate residual stones and encrustations, thereby improving the efficiency of radiologists in reviewing images.

## Data Availability

The original contributions presented in the study are included in the article/Supplementary Material, further inquiries can be directed to the corresponding author.
