# Supplementary material for: A method for detecting ureteral stent encrustations in medical CT images based on Mask-RCNN and 3D morphological analysis
Source: Front Physiol. 2024 Aug 29;15:1432121. doi: 10.3389/fphys.2024.1432121 (PMC11394187; doi:10.3389/fphys.2024.1432121)
Supplement: Supplementary file 1 [file DataSheet1.docx]

1. miniDL module


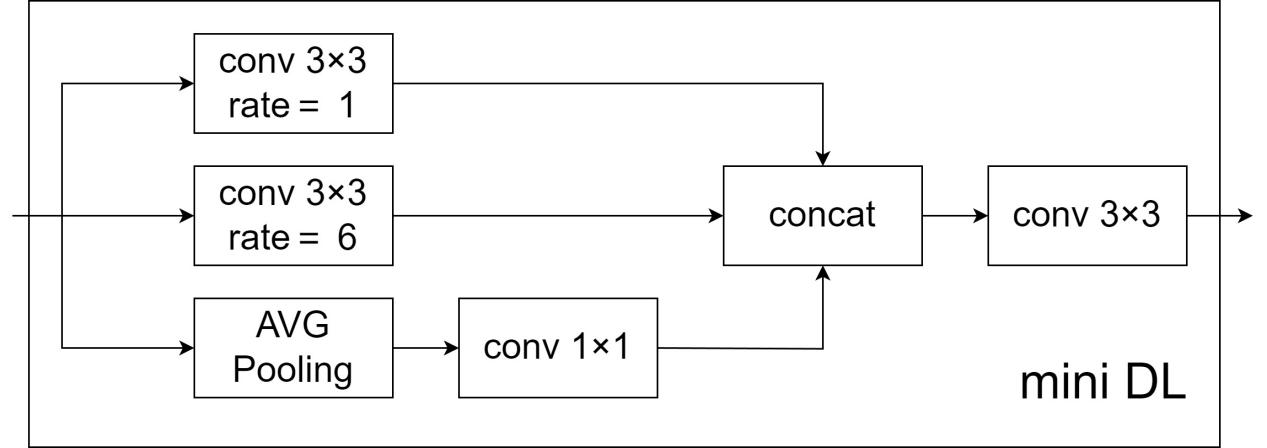


In the decoder of the instance segmentation task, we follow the design philosophy of Mask-RCNN, taking the four-layer features of different scales from Res34-FPN as input. With predefined sizes,it can generate anchors centered on the available spatial locations. The Regional Proposal Network (RPN) transforms each anchor to obtain a new bounding box and a bounding box confidence score, which evaluates the effectiveness of the anchor, and determines the anchors containing detection targets, followed by non-maximum suppression (NMS) to filter out bounding boxes with overlap rates exceeding a certain threshold, thereby determining the Region of Interest (ROI).

2.Parameter design

The feature extraction module utilizes the pre-trained weights from the ImageNet-pretrained ResNet-34 in the PyTorch deep learning framework to initialize the network. The network is trained using the SGD optimization method, with an initial learning rate of 0.01, momentum of 0.9, and weight decay of 0.0001. The learning rate is adjusted during training using the multistep approach, with a total of 85 epochs. The learning rate is reduced by a factor of 10 at two nodes, specifically at the 20th and 50th epochs.

At the beginning of training, we also have a warm-up stage for the learning rate, where the learning rate is linearly increased from 0.001 to 0.01 over the first 200 batches. In the initial settings for the instance segmentation subtask, the number of anchors generated is set differently for the training and validation sets. For the training set, 1200 anchors are initially generated, and after non-maximum suppression, 200 anchors are retained. For the validation set, 600 and 100 anchors are generated, respectively. The initial anchor size is set to 2, and the aspect ratio of the anchor is set to 5 types: (0.2, 0.5, 1, 2, 5). The non-maximum suppression threshold is set to 0.5. During training, the network takes a complete image as input, with a size of 512x512, and is randomly flipped horizontally with a probability of 0.5. The image is also randomly scaled within the range of [0.5, 2.0] by sampling from the original image.

3.Loss function design

Unlike the loss function for semantic segmentation, instance segmentation is a multi-output network that includes a target detection module and a simple semantic segmentation branch for mask prediction. For each ROI, in addition to the mask loss, which can still be calculated using the pixel-wise cross-entropy loss function as described in Chapter 3, the losses for bounding box regression and target classification also need to be considered.
